# Culture Impacts the Neural Response to Perceiving Outgroups Among Black and White Faces

**DOI:** 10.3389/fnhum.2019.00143

**Published:** 2019-05-01

**Authors:** Colleen Hughes, Laura G. Babbitt, Anne C. Krendl

**Affiliations:** ^1^Department of Psychological and Brain Sciences, Indiana University, Bloomington, IN, United States; ^2^Department of Economics at Tufts University, Medford, MA, United States

**Keywords:** culture, race perception, functional magnetic resonance imaging, stigma, bias, cultural learning

## Abstract

Outgroup members (e.g., individuals whose racial identity differs from perceivers’) are stigmatized in Eastern and Western cultures. However, it remains an open question how specific cultural influences affect stigmatization. In this study, we assessed whether cultural learning (i.e., social information acquired from the people in one’s environment) associated with Chinese individuals’ relocation to the United States differentiated the response to multiple outgroups. Two types of cultural learning predict diverging responses to outgroups – awareness of stereotypes about different racial outgroups is associated with increased negative affect and cognitive control toward the stereotyped outgroup. Conversely, intergroup contact attenuates those responses, and does so to a greater extent for individuals from Western cultures. As Chinese–Americans would have had more opportunities to have contact with both White and Black individuals (relative to the Chinese participants), we explored their responses to outgroups as well. Because the neural regions associated with stereotyping and intergroup contact have been well-characterized, we used neuroimaging to disentangle these possibilities. Eighteen White American, 18 Chinese–American, and 17 Chinese participants – who had relocated to the United States less than 1 year prior – viewed images of Black and White individuals while undergoing functional magnetic resonance imaging (fMRI). Participants also completed measures of awareness of cultural stereotypes in the United States about Black and White individuals, implicit bias, and experiences with White and Black individuals. Behaviorally, White American and Chinese–American participants had more intergroup contact with either race than did Chinese participants, but there was no effect of participant group on stereotype knowledge or implicit bias. When viewing faces of White (as compared to Black) individuals while undergoing fMRI, White American (relative to Chinese) participants had attenuated activation in regions of the brain associated with cognitive control, including the right dorsolateral prefrontal cortex, dorsal striatum, and ventrolateral prefrontal cortex. Chinese–Americans’ neural response to either race did not differ from White American or Chinese participants. Taken together, outgroup biases seemed to emerge in a culturally-dependent way based on variability in intergroup contact, but not necessarily awareness of stereotypes.

## Introduction

Stigma – a trait or condition that makes an individual devalued in certain contexts – is associated with robust and pervasive bias in both Eastern and Western cultures (Cuddy et al., 2009). Prior work has examined responses toward culture-specific stigmatized groups (e.g., elderly, homeless) between individuals from Eastern and Western cultures (Krendl, 2016). However, it remains an open question to what extent culture contributes to stigmatization. That is, how does culture affect different factors (e.g., stereotypes, interpersonal experiences) that underlie responses toward outgroups? Emerging research on cultural differences has focused on identifying similarities and differences in how individuals respond to groups that are stigmatized in their respective cultures. The current study explores how individuals from Eastern cultures acquire stigmas (e.g., about Black compared to White individuals) when they have relocated to a new cultural context. Understanding the impact of culture is important because it can provide valuable insight into the mechanisms by which negative biases toward outgroups emerge.

Recent research suggests that individuals in Eastern and Western cultures stigmatize certain groups (e.g., homeless individuals) to a similar extent, but differ in the extent to which they stigmatize groups whose social status differs in the two cultures (e.g., older adults; Krendl, 2016). One reason for this might be cultural learning – social information or responses acquired from the people in one’s environment (Tomasello et al., 1993). We focused on the influence of two types of cultural learning that may differentiate responses to multiple outgroups: stereotypes and interpersonal experiences. If individuals in Eastern and Western cultures attribute different stereotypes to the same outgroup members, then those different stereotypes will have disparate effects on behavior in each culture (e.g., Cuddy et al., 2007). However, one study found that stereotypes about stigmatized groups are largely consistent across cultures (i.e., American, European, and East Asian cultures; Cuddy et al., 2009). Another possibility is that stereotypes may affect behavior differently across cultures depending on an individual’s interpersonal experiences with outgroup members (i.e., intergroup contact). Intergroup contact – the amount or quality of interpersonal experiences one has with outgroup members (Islam and Hewstone, 1993) – has been widely shown to reduce prejudice (Pettigrew and Tropp, 2006). Specifically, intergroup contact reduces reliance on stereotypes (Rothbart and John, 1985) by promoting less stereotypic (Kawakami et al., 2000) and more heterogenous cognitive representations of outgroup members (Wolsko et al., 2003). For instance, Pauker et al. (2018) found that White students who moved from the continental United States (which is relatively racially homogeneous) to Hawaii (which is racially heterogeneous) had decreased levels of prejudice, particularly as a function of more intergroup contact (Pauker et al., 2018). In this way, intergroup contact represents another form of learning about outgroup members within cultures (e.g., among racial groups in the United States; Shelton, 2003) and across cultures (e.g., among international students and individuals from their host countries; Ward and Kennedy, 1993).

A recent meta-analysis examined the effectiveness of intergroup contact in reducing prejudice across different cultures (Kende et al., 2018). The study found that contact attenuated prejudice to a greater extent in Western than in Eastern cultures (Kende et al., 2018). There are several reasons why that might be. First, the overall magnitude and scope of how prejudice develops may differ in Eastern and Western cultures. That is, cultural learning about the negative stereotypes associated with specific groups may differ in the two cultures. On the other hand, individuals in the two cultures may differ in how they respond to contact. In Eastern cultures, individuals tend to endorse collectivist values that emphasize the role of the self within his/her groups (Triandis, 1988), which may make it difficult to see other groups as equal in an intergroup situation. Individuals in Western cultures, however, tend to endorse individualistic values that emphasize the role of the self as independent of groups (Markus and Kitayama, 1991). Individuals in Western cultures may thus benefit more from intergroup contact because it may be relatively less difficult for them to see other groups as equal than are individuals in Eastern cultures (Triandis and Trafimow, 2008).

In the current study, we used neuroimaging order to disentangle the impact of culturally-learned stereotypes and intergroup contact on Eastern and Western individuals’ responses to outgroups. Neuroimaging provides valuable insight into this question because the neural correlates associated with prejudice are well-characterized (e.g., Amodio, 2014). For instance, several neuroimaging studies with White participants in the United States found that negative culturally-learned stereotypes about Black individuals differentiated the neural response in the amygdala to Black versus White targets (Phelps et al., 2000; Lieberman et al., 2005). Other work has found that awareness of stereotypes elicits activation in the oribitofrontal cortex (OFC) in White American and Chinese participants (Freeman et al., 2015). These regions have been linked to affective aspects of prejudice (Amodio, 2014), which may reflect the negative affective content of stereotypes about stigmatized groups in particular (e.g., Black vs. White individuals in the United States; Kawakami et al., 2000).

Intergroup contact, on the other hand, is related to neural activity associated with cognitive control (e.g., Walker et al., 2008). A number of prior studies have found increased neural activation in prefrontal cortex regions associated with cognitive control (e.g., lateral prefrontal cortex, anterior cingulate cortex) that seem to play a role in regulating perceivers’ automatic responses toward stigmatized individuals (Beer et al., 2008; Krendl et al., 2012; Cassidy et al., 2016). These findings support other work showing that perceiving and interacting with outgroup members is cognitively demanding (Richeson et al., 2003; Richeson and Trawalter, 2005). Conversely, attenuated activity in these regions is related to intergroup contact. For example, two neuroimaging studies with United States-born White participants found that childhood contact with Black individuals was associated with attenuated activation of neural regions associated with cognitive control [e.g., dorsolateral prefrontal cortex (dlPFC)] but also awareness of stereotypes (e.g., OFC) when viewing faces of Black individuals (Cloutier et al., 2014, 2016). Similarly, the length of time that Asian immigrants spent in Europe was associated with decreased amygdala and OFC activation in response to emotional White (i.e., outgroup) faces (Derntl et al., 2009, 2012). The current work thus builds on past work by examining two types of cultural learning (stereotype knowledge and intergroup contact) about multiple outgroups (Black and White individuals) in a cross-cultural sample to provide a comprehensive assessment of their contributions to the emergence of outgroup biases.

The current study examines the extent to which cultural learning affects how stereotype knowledge and/or intergroup contact contribute to outgroup bias. To do this, we recruited Chinese participants who had recently relocated to the United States and White American participants to complete a neuroimaging study in which they viewed images of Black and White individuals. We focused on responses to Black and White individuals because both were relatively novel outgroups for Chinese participants, but the former is associated with robust negative stereotypes in the United States. Extant work with White Americans had identified distinct neural components that are engaged in response to evaluating Black (versus White) faces that relate to stereotype knowledge and intergroup contact, respectively (for review, see Amodio, 2014). Therefore, we expected that the neural responses to Black versus White faces would differ between groups to the extent that Chinese participants were unfamiliar with stereotypes about these groups or had lesser intergroup contact with them compared to White Americans.

To further interrogate this question, we also included a group of first-generation Chinese–American participants. We included this group for two reasons. First, Chinese participants may have similar stereotype knowledge about Black individuals as do White American participants, but having similar stereotype knowledge does not necessarily lead to similar levels of prejudice (e.g., Devine, 1989). Chinese–Americans were exposed to similar cultural prejudices toward Blacks across their lifetime as White American participants. As such, this group allowed us to consider the impact of cultural learning on stereotype knowledge as well as the strength of stereotypic associations (i.e., prejudice). The second reason we included this group is that Chinese participants may have different intergroup contact due to the duration of their time in the United States, rather than cultural differences between Eastern and Western cultures. Put another way, Chinese–Americans may be influenced by Eastern cultural values that moderate the influence of contact effects, but have the same amount and quality of intergroup contact with White and Black individuals as White Americans. Supporting this notion, Pornpattananangkul et al. (2016) found that collectivist cultural values are evidenced most strongly amongst native Japanese individuals and to a lesser extent among Japanese–American individuals; whereas both groups had higher endorsement of these values than White American individuals. Because Chinese–Americans would have had more opportunities to have contact with both White and Black individuals (relative to the Chinese participants), we explored whether contact affected their response to outgroup members.

We examined cultural learning about multiple outgroups by measuring (a) awareness of stereotypes and (b) intergroup contact among all three groups of participants. Behaviorally, we hypothesized that White American and Chinese–American participants would have greater awareness of racial stereotypes about Black and White groups than Chinese participants (Hypothesis 1A). Because stereotypes of Blacks (vs. Whites) are more negative in American culture (Kawakami et al., 2000), we correspondingly predicted that both White American and Chinese–American participants would have higher implicit bias toward Blacks than do Chinese participants. We also predicted that White American and Chinese–American participants would have a greater amount and quality of intergroup contact with White and Black individuals compared to Chinese participants (Hypothesis 1B). On a neural level, we tested two possibilities that were not mutually exclusive. First, if Chinese participants did not have similar stereotype knowledge about these groups as did White Americans, then they may not show increased neural response in regions associated with culturally-learned negative stereotypes (e.g., OFC, amygdala) to Black (as compared to White) targets (Hypothesis 2A). Second, if there are cultural differences in how contact affects how novel faces are perceived, then we would expect Chinese participants – who would be less likely to have contact with both outgroups – to not have higher activation in neural regions associated with cognitive control (e.g., dlPFC) when perceiving Black versus White faces, unlike White Americans (Hypothesis 2B). Chinese–American participants may have a similar pattern as White Americans, but to a lesser extent (Hypothesis 2C). Finally, we explored the possibility that group differences in neural activity would correspond with individual differences in stereotype knowledge or intergroup contact. To reduce the likelihood of false positives, we limited the number of correlations tested by focusing on relating behavioral and neural measures where differences between the groups emerged.

## Materials and Methods

### Participants

Three groups of participants were recruited for the current study. The original sample was comprised of 19 White American, 19 Chinese–American, and 18 Chinese participants. This sample size was selected to ensure sufficient power for our analyses (Desmond and Glover, 2002) and is similar to samples in other cross-cultural neuroimaging work (Chiao et al., 2008; Derntl et al., 2012; Pornpattananangkul et al., 2016). One White American participant was excluded for not completing the imaging session (due to claustrophobia). Two participants (1 Chinese–American, 1 Chinese) were excluded from analyses because they moved more than 2 mm during the task of interest. The final sample included 18 White American (eight female, *M_age_* = 20.88, *SD* = 1.11), 18 Chinese–American (nine female, *M_age_* = 20.22, *SD* = 1.44), and 17 Chinese (11 female, *M_age_* = 21.71, *SD* = 2.54) participants.

#### Participant Recruitment

Participants were recruited from the greater Boston area by two Mandarin-speaking research assistants via electronic mailings and flyers. Chinese participants were international university students who were studying in the United States. Most of these individuals were born in mainland China (*N* = 15). The remaining were born in Singapore or Taiwan. These individuals all reported on recruitment that they had been in the United States for less than 1 year. First-generation Chinese–American participants were university students born in the United States to parents who had been born in China. White American participants were also university students, born in the United States to parents who had also been born and raised in the United States. Special attention was paid to matching the participants for age, and efforts were made to control for gender as well.

### Procedure

#### Behavioral Methods

While undergoing functional magnetic resonance imaging (fMRI), participants completed several other tasks outside the scope of this work (e.g., see Krendl, 2016). The order of the tasks was counterbalanced across participants. During the race perception task, participants viewed images of Black or White male or female faces with neutral expressions one at a time on screen. The faces were similar ages across conditions. In total, participants viewed 160 images (40 per condition; 80 male faces in total). All images were 2.78 inches in height, 72 pixels/inch resolution, in grayscale, and cropped into an oval directly around the face removing the hair, shoulders, and background of the original image. The images were presented on either the left or right side of the screen, and participants were asked to indicate via keypress the side of the screen on which the image appeared. The female faces were included for a separate research question, and were not analyzed here. We focused on male faces because stereotypes about Black individuals have been previously shown to be more descriptive of men than women (Eagly and Kite, 1987). This approach is consistent with prior work showing that the perceptions of different groups varied by gender (Johnson et al., 2012).

Following the scan, participants completed several questionnaires that assessed their attitudes toward and experience with Black and White individuals, as well as measures of acculturation to the United States. Chinese participants were given the choice of completing the survey items in English or Mandarin. We measured participants’ explicit knowledge of stereotypes about Black and White individuals using a measure adapted from Goff et al. (2008). Participants responded whether they were aware of seven traits about Black and White individuals, respectively, by making a yes or no response. Of these, four per target race were stereotypes of these groups (e.g., “I am aware of the stereotype that Black Americans are athletic”) and three per target race were not stereotypes of these groups (e.g., “I am aware of the stereotype that Black Americans like to whisper”). The number of “yes” responses were totaled separately for the four Black and White stereotypes to create measures of stereotype awareness about each group. Responses to the traits that were not stereotypical of the target groups were not analyzed. Participants also completed the Internal (IMS; Cronbach’s α = 0.85) and External (EMS; Cronbach’s α = 0.80) Motivation to Control Prejudice Scales (Plant and Devine, 1998). These measures assessed the participants’ motivations to behave in a non-prejudiced manner toward Black individuals. Responses to the items for each scale were averaged. The final racial prejudice measure they completed was the Implicit Association Test (IAT; Greenwald et al., 1998, 2003), which is a widely-used measure of implicit racial attitudes. The IAT in the current study used the target categories (“Black” and “White”) and descriptor categories (i.e., “Good” and “Bad”). Ten Black male faces and 10 White male faces were used in the task, along with 10 positive (e.g., “love”) and negative (e.g., “foul) words. The IAT was scored using standardized reaction time differences as in prior work (Greenwald et al., 2003) where higher scores indicate higher implicit bias toward Black versus White individuals. Participants were randomly assigned to one of four versions that counterbalanced the order of the pairings of the categories and the side of the screen on which the pairings appeared – neither of which affected the IAT score, *F*s < 2.99, *p*s > 0.09.

Participants completed the Intergroup Contact Scale (Islam and Hewstone, 1993), which assesses previous experiences (i.e., quantity and quality) with Black individuals whose gender was not specified. Quantity of contact referred to the number or amount of experiences with Black individuals (e.g., “In the past, I have rarely interacted with Black people”), whereas quality of contact referred to the number of positive interactions with Black individuals (e.g., “Over the course of my life, I have had many Black friends”). We also modified the items to assess their previous experiences with White individuals by replacing the word “Black” in the question items with “White” (e.g., “The neighborhood(s) I grew up in had mostly White people”). Participants responded to four items about amount of contact with White individuals and two items about Black individuals, and three items per group measuring quality of contact. Responses were scored on a 1 (*strongly disagree*) to 7 (*strongly agree*) Likert scale. The reliabilities for the contact scales were good (Cronbach’s αs = 0.69–0.80). The items for each subscale were averaged to create composite measures of these constructs. All participants also completed the East Asian Acculturation Measure (EAAM; Barry, 2001) – a 29-item scale that includes four subscales that assess assimilation, separation, integration, and marginalization. These four subscales assess the extent to which East Asians are: willing to forgo their own cultural identity in order to integrate with their new society (assimilation; Cronbach’s α = 0.86); maintain their ethnic identity and traditions without incorporating their new culture into their identity (separation; Cronbach’s α = 0.81); maintain their ethnic identity while also embracing traditions of their new culture (integration; Cronbach’s α = 0.49); and feel as though they have no cultural or psychological connection with either their traditional or current culture (marginalization; Cronbach’s α = 0.86). As the integration subscale had poor reliability, it was not analyzed. The items for each subscale were summed to create composite measures of these constructs.

#### fMRI Methods

Anatomical and functional whole-brain imaging was performed on a 3.0 T Siemens Trio Scanner (Trio, Siemens, Ltd., Enlargen, Germany) using standard data acquisition protocols. Anatomical images were acquired using a high-resolution 3D magnetization prepared rapid gradient echo sequence (MP-RAGE; 144 sagittal slices, TE = 7 ms, TR = 2200 ms, flip angle = 7°, 1 mm × 1 mm × 0.89 mm voxels). Functional images were collected in one functional run of 172 time points, using a fast field echo-planar sequence sensitive to blood-oxygen level-dependent contrast (T2^∗^) (31 axial slices per whole-brain volume, matrix: 72 × 72, resolution (xyz): 3 mm × 3 mm × 4, 0 mm skip, TR = 2000 ms). Data underwent standard preprocessing in SPM12 (Wellcome Trust Centre for Neuroimaging, London, United Kingdom ^1^) to remove sources of noise and artifact. Here, images were realigned to correct for motion, normalized to the MNI (Montreal Neurological Institute) template, and smoothed using an 6 mm FWHM isotropic Gaussian kernel. Two White American participants did not have anatomical images and thus were registered during preprocessing to the single subject T1 image from the MNI-152 data included in SPM.

To examine cultural differences, we used a general linear model incorporating task effects for the four different image types (Black male, Black female, White male, White female), the two focal participant groups (White American, Chinese), and covariates of no interest (a session mean, a linear trend, and six movement parameters derived from realignment corrections) to compute parameter estimates (β) and t-contrast images (containing weighted parameter estimates) for each comparison at each voxel and for each subject. Unless otherwise noted, imaging data were extracted at a threshold of *p* < 0.05, corrected. A Monte Carlo conversion script from Slotnick et al. (2003) determined the extent threshold required to convert *p* < 0.005 uncorrected to *p* < 0.05 corrected (e.g., Lieberman and Cunningham, 2009). We chose 1000 iterations of the Monte Carlo to select the most conservative threshold (18 contiguous voxels at *p* < 0.005; for a discussion on cluster thresholding, see Woo et al., 2014). We conducted region of interest (ROI) analyses by extracting parameter estimates for each condition versus a fixation baseline using the MarsBar ROI toolbox for SPM (6 mm sphere from peak activations; Brett et al., 2002).

## Results

**Hypothesis 1:** White Americans and Chinese–Americans had greater quantity and quality of contact with Black and White individuals than Chinese participants.

See Table 1 for means and standard deviations for all individual difference measures. The variances on the individual difference measures among the participant groups were, in some cases, not equal – that is, the variances were significantly different based on Levene’s test for equality of variances. When this occurred, we used a Welch’s *t*-test for *post hoc* comparisons that did not use the pooled variances and applied a correction to the degrees of freedom (see Welch, 1947).

**Table 1:** Individual difference measures by participant group.

	White American	Chinese–American	Chinese
Intergroup contact with white	5.13 (0.46)^a^	4.38 (1.14)^b^	3.10 (1.09)^c^
Intergroup contact with black	5.00 (1.36)^a^	4.22 (1.19)^b^	2.91 (1.28)^c^
Positive intergroup contact with white	6.31 (0.49)^a^	6.00 (0.67)^a^	4.59 (0.94)^b^
Positive intergroup contact with black	5.09 (1.03)^a^	4.44 (1.19)^a^	4.22 (1.46)^b^
Stereotype knowledge about white	3.11 (1.02)	2.89 (1.02)	2.24 (1.25)
Stereotype knowledge about black	3.78 (0.73)	3.83 (0.38)	3.35 (1.06)
Black-white race IAT	0.41 (0.27)	0.39 (0.56)	0.66 (0.44)
Internal motivation to control prejudice	5.63 (1.12)	5.24 (1.28)	4.96 (1.30)
External motivation to control prejudice	3.79 (1.17)	3.46 (1.59)	3.83 (1.23)
Assimilation	41.17 (5.74)^a^	39.84 (8.69)^a^	24.28 (7.17)^b^
Separation	17.00 (3.38)^a^	23.95 (9.28)^b^	28.83 (6.07)^b^
Marginalization	23.56 (8.69)	26.58 (12.41)	27.83 (8.15)

*Mean (standard deviation). Different subscripts in each row indicate differences between the samples based on t-test comparisons reported in-text.*

### Stereotype Knowledge and Implicit Racial Bias

We tested for group differences using a univariate ANOVA (Group: White American, Chinese–American, and Chinese) with measures for Black and White individuals entered as separate dependent variables. There was no main effect of group on participants’ knowledge of stereotypes about Black or White individuals, *F*s < 2.97, *p*s > 0.06; IMS or EMS, *F*s < 1.44, *p*s > 0.24.

Similarly, there was no main effect of group on participants’ implicit racial bias using standardized reaction time difference scores (Greenwald et al., 2003), *F*(2,53) = 2.09, *p* = 0.14. However, these scores reflect the strength of negative associations about Black individuals relative to the strength of negative associations about White individuals, based on the respective blocks where those categories are paired with negative words. Given that both Black and White individuals are outgroup members to the Chinese participants, we also wanted to assess the strength of negative associations toward these groups individually to determine whether stronger bias toward one group or the other drove their overall bias score. To do this, we conducted a mixed measures ANOVA on the raw reaction times from the IAT with group as a between-subjects factor and block (Black bias: [White-Good, Black-Bad], White bias: [White-Bad, Black-Good]) as a within-subjects factor. There was an interaction between group and block, *F*(2,50) = 5.39, *p* = 0.008, $\eta_{p}^{2}$ = 0.18. We tested for group differences in each block using a univariate ANOVA (Group: White American, Chinese–American, and Chinese). There was a main effect of group on reaction times reflecting the strength of negative associations about White individuals (White bias block), *F*(2,50) = 5.25, *p* = 0.009, $\eta_{p}^{2}$ = 0.17; but not Black individuals (Black bias block), *F*< 1. Specifically, Chinese participants (*M* = 898.64, *SD* = 238.71) had less anti-White bias (e.g., slower reaction times associating Whites with negative words than did White American: *M* = 742.29, *SD* = 127.60 or Chinese–American: *M* = 736.71, *SD* = 109.78 participants, *t*s > 2.44, *p*s < 0.02; who did not differ from each other, *t* < 1).

### Intergroup Contact

We tested for differences in amount and quality of intergroup contact, separately, using a mixed measures ANOVA with group (White American, Chinese–American, Chinese) as a between-subjects factor and target race (White, Black) as a within-subjects factor. For amount of intergroup contact, there was no main effect of target race, *F* < 1; or interaction, *F* < 1. However, there was a main effect of group on the amount of intergroup contact, *F*(2,50) = 40.58, *p* < 0.001, $\eta_{p}^{2}$ = 0.62. *Post hoc* tests revealed that White American participants (*M* = 5.99, *SD* = 0.66) had more intergroup contact overall (White *and* Black individuals) than did either Chinese–American (*M* = 5.19, *SD* = 0.65), *t*(34) = 3.65, *p* = 0.001, *d* = 1.22, 95% CI [0.51, 1.93]; or Chinese participants (*M* = 3.20, *SD* = 0.89), *t*(33) = 10.65, *p* < 0.001, *d* = 3.58, 95% CI [2.51, 4.64]. Chinese–American participants also had more contact with both racial outgroups than Chinese participants, *t*(33) = 7.62, *p* < 0.001, *d* = 2.65, 95% CI [1.67, 3.46].

Regarding the quality of intergroup contact with White and Black individuals, there was no interaction, *F*< 1. However, there were main effects of group, *F*(2,50) = 25.25, *p* < 0.001, $\eta_{p}^{2}$ = 0.50, and of target race, *F*(2,50) = 59.88, *p* < 0.001, $\eta_{p}^{2}$ = 0.55. *Post hoc* tests for the main effect of group found that White Americans (*M* = 5.70, *SD* = 0.66) had more positive intergroup contact overall than Chinese–Americans (*M* = 5.22, *SD* = 0.73), *t*(34) = 2.07, *p* = 0.046, *d* = 0.69, 95% CI [0.02, 1.36]; or Chinese participants (*M* = 3.90, *SD* = 0.91), *t*(33) = 6.71, *p* < 0.001, *d* = 2.28, 95% CI [1.42, 3.13]. Chinese–American participants also had more positive contact with White and Black individuals than Chinese participants, *t*(33) = 4.73, *p* < 0.001, *d* = 1.60, 95% CI [0.84, 2.37]. For the main effect of target race, all groups had more positive contact with White (*M* = 5.65, *SD* = 1.03) than Black (*M* = 4.27, *SD* = 1.44) individuals.

### Acculturation

To account for multiple comparisons across the three EAAM subscales, we applied a Bonferroni correction that resulted in a new threshold of *p* < 0.017 to reach significance for the group-wise ANOVA. There was a main effect of group on two key dimensions of acculturation: assimilation, *F*(2,50) = 28.39, *p* < 0.001, $\eta_{p}^{2}$ = 0.53; and separation, *F*(2,50) = 13.15 *p* < 0.001, $\eta_{p}^{2}$ = 0.35. White American and Chinese–American participants did not differ in their level of assimilation, *t* < 1; however, both reported more assimilation than Chinese participants, *t*s > 5.77, *p*s < 0.001. In contrast, White American participants reported less separation than both Chinese–American and Chinese participants, *t*s > 2.76, *p*s ≤ 0.009; who did not differ from each other, *t*(33) = 1.97, *p* = 0.06, *d* = 0.66, 95% CI [−0.02, 1.34]. There was no main effect of group on the marginalization subscale of the EAAM, *F* < 1.

**Hypothesis 2:** The Neural Response to White, But Not Black Faces, Varied by Participant Group.

**FIGURE 1:**
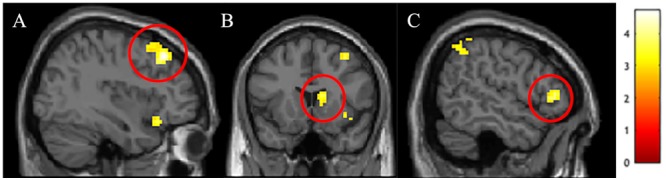
Activations in **(A)** right dorsolateral prefrontal cortex (dlPFC), **(B)** right dorsal striatum, and **(C)** right ventrolateral prefrontal cortex (vlPFC) that were stronger in White Americans than Chinese participants for the Black male faces > White male faces *t*-contrast.

We conducted between-group *t*-tests between White American and Chinese participants across the whole brain to identify cultural differences in the differential neural response to outgroup male faces. Several regions associated with cognitive control – right dlPFC, right vlPFC, and right dorsal striatum – were more active for White American participants (versus Chinese participants) when perceiving Black (vs. White) faces (see Table 2 for a complete list of activations; Figure 1). ROI analyses were conducted to better characterize group-wise differences in perceiving outgroups in these regions (see section “Materials and Methods”). Parameter estimates from the ROI analyses were entered into a 3 (Group: White American, Chinese–American, Chinese) × 2 (Target Race: Black male faces, White male faces) ANOVA with target race as a repeated measure. We included Chinese–Americans (Hypothesis 2C) in the ROI analyses to better characterize whether the differences observed related to culture (shared with Chinese participants) or intergroup contact (shared with White Americans).

**Table 2:** Results from the whole-brain *t*-contrasts between White American and Chinese participants when they viewed Black male faces versus White male faces, *p* < 0.05 corrected (*p* < 0.005 uncorrected, *k* = 18).

Region	BA	*k*	*t*	MNI coordinates
**White American > Chinese**				
R dorsolateral prefrontal cortex	8/9	127	4.72	39, 30, 45
	8	^∗^	3.54	36, 15, 51
R superior frontal gyrus	8	24	4.21	12, 33, 48
		^∗^	3.57	15, 24, 45
R dorsal striatum	48	27	4.19	9, 12, 12
R ventrolateral prefrontal cortex	45	42	3.87	54, 33, 6
	44	^∗^	3.28	60, 24, 9
	45	^∗^	2.97	60, 27, 0
R insular cortex	13	46	3.66	36, 18, −9
	47	^∗^	3.56	39, 24, −18
	13	^∗^	3.04	27, 21, −12
R inferior parietal lobule	39	53	3.52	57. −57, 48
	7	^∗^	3.47	51, −45, 63
	39	^∗^	3.42	51, −54, 57
**Chinese > White American**				
*No significant clusters*				

*^∗^Sub-cluster of above-listed region.*

### Right dlPFC

**FIGURE 2:**
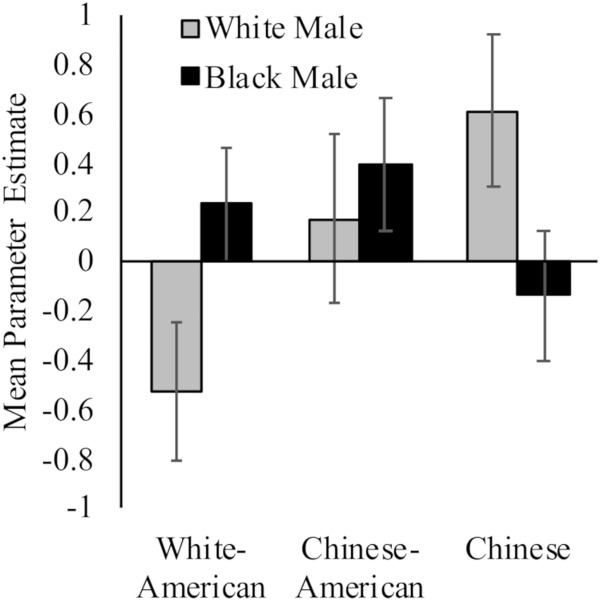
Neural activation in right dlPFC in response to perceiving White and Black male faces. Graph show mean parameter estimates in this region for each group when they viewed images of White and Black male faces as compared to a fixation baseline. Error bars are ±1 standard error of the mean.

While White Americans had less activation toward White (*M* = −0.53, *SD* = 1.19) versus Black (*M* = 0.23, *SD* = 0.99) faces, *t*(17) = 2.91, *p* = 0.01, *d* = 0.69, 95% CI [0.02, 1.37]; Chinese participants demonstrated the opposite pattern (*M_White_* = 0.61, *SD* = 1.27; *M_Black_* = −0.14, *SD* = 1.07; *t*(16) = 3.98, *p* = 0.001, *d* = 0.64, 95% CI [−0.05, 1.33]). Chinese–Americans did not have differential activation in right dlPFC to White (*M* = 0.17, *SD* = 1.47) and Black faces (*M* = 0.39, *SD* = 1.16) faces, *t* < 1. Moreover, the differences between groups were driven by neural response to perceiving White faces, *F*(2,50) = 3.35, *p* = 0.04, $\eta_{p}^{2}$ = 0.12; rather than Black faces, *F*(2,50) = 1.11, *p* = 0.34. See Figure 2.

### Right Dorsal Striatum

**FIGURE 3:**
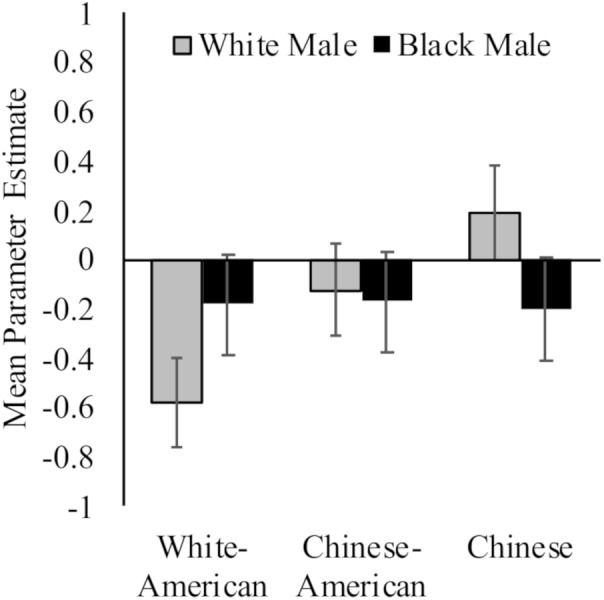
Neural activation in right dorsal striatum in response to perceiving White and Black male faces. Graph show mean parameter estimates in this region for each group when they viewed images of White and Black male faces as compared to a fixation baseline. Error bars are ±1 standard error of the mean.

While White Americans had less activation toward White (*M* = −0.58, *SD* = 0.71) versus Black (*M* = −0.18, *SD* = 0.68) faces, *t*(17) = 2.94, *p* = 0.009, *d* = 0.58, 95% CI [−0.09, 1.24]; Chinese participants again demonstrated the opposite pattern (*M_White_* = 0.19, *SD* = 0.71; *M_Black_* = −0.20, *SD* = 0.68; *t*(16) = 3.91, *p* = 0.001, *d* = 0.56, 95% CI [−0.12, 1.25]). Chinese–Americans did not have differential activation in right dorsal striatum to White (*M* = −0.12, *SD* = 0.91) and Black (*M* = −0.17, *SD* = 0.71) faces, *t* < 1. Group differences were driven by neural response to perceiving White faces, *F*(2,50) = 4.24, *p* = 0.02, $\eta_{p}^{2}$ = 0.15; rather than Black faces, *F* < 1. See Figure 3.

### Right vlPFC

**FIGURE 4:**
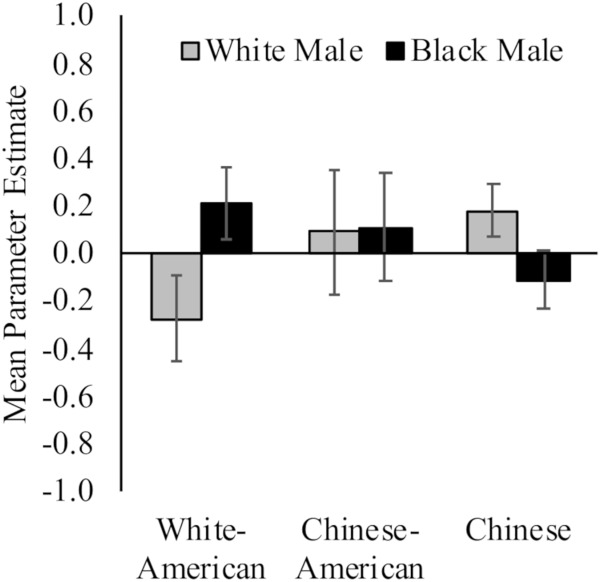
Neural activation in right vlPFC in response to perceiving White and Black male faces. Graph show mean parameter estimates in this region for each group when they viewed images of White and Black male faces as compared to a fixation baseline. Error bars are ±1 standard error of the mean.

White Americans had less activation toward White (*M* = −0.28, *SD* = 0.76) versus Black (*M* = 0.21, *SD* = 0.64) faces, *t*(17) = 3.61, *p* = 0.002, *d* = 0.70, 95% CI [0.02, 1.37]; and Chinese participants demonstrated the opposite pattern (*M_White_* = 0.18, *SD* = 0.44; *M_Black_* = −0.11, *SD* = 0.50; *t*(16) = 2.13, *p* = 0.05, *d* = 0.62, 95% CI [0.07, 1.30]). Chinese–Americans did not have differential activation in right vlPFC to White (*M* = 0.09, *SD* = 1.11) and Black (*M* = 0.11, *SD* = 0.95) faces, *t* < 1. Yet, there were no differences between the groups in ventrolateral prefrontal cortex (vlPFC) activation to White or Black faces, *F*s < 1.54, *p*s > 0.22. See Figure 4.

Although effects of target gender were outside the scope of the current paper, we tested and found that no significant main effects of group or target race, *F*s < 1.51, *p*s > 0.23; or interactions emerged when running the same ROI analyses using neural activity to female faces in the right dlPFC, dorsal striatum, or vlPFC, *F*s < 1.

### Independently-Defined ROI Analysis

To characterize whether the regions identified from the contrast were related to cognitive control, we extracted parameter estimates for each target race using unbiased peaks of activation identified from other research. Peaks in the right dlPFC, right vlPFC, and right dorsal striatum were identified from Kelly et al. (2004) as being involved in response inhibition, a core aspect of cognitive control (Miyake et al., 2000). The interaction between group and target race was not significant for the independently-defined right vlPFC or right dorsal striatum ROIs, *F*s < 1.54, *p*s > 0.22. Only the right dlPFC demonstrated an interaction between group and target race (male faces): *F*(2,50) = 3.68, *p* = 0.03, $\eta_{p}^{2}$ = 0.13. Specifically, White Americans had less activation toward White (*M* = −0.33, *SD* = 0.92) versus Black (*M* = 0.06, *SD* = 0.67) faces, *t*(17) = 2.15, *p* = 0.046, *d* = 0.48, 95% CI [0.18, 1.15]. Chinese–American (*M_white_* = −0.22, *SD* = 0.98; *M_black_* = −0.16, *SD* = 0.84) and Chinese (*M_white_* = 0.39, *SD* = 0.85; *M_black_* = 0.10, *SD* = 0.54) participants did not differ in their activation in right dlPFC to White male faces, *t*s < 1.58, *p*s > 0.13. Although not reaching statistical significance, the differences between groups appeared to be related to perceiving White faces, *F*(2,50) = 3.04, *p* = 0.057, $\eta_{p}^{2}$ = 0.11; rather than Black faces, *F* < 1.

### Positive Intergroup Contact Is Correlated With an Attenuated Neural Response to White Faces Among White American Participants

**FIGURE 5:**
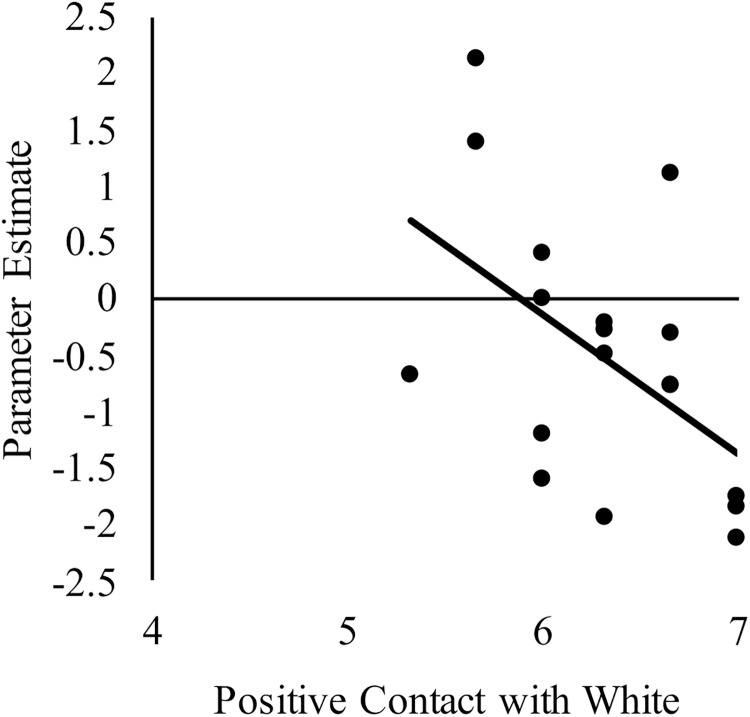
Correlation between White American’s positive contact with White individuals (ingroup) and the neural activation in right dlPFC to perceiving White male (ingroup) faces compared to a fixation baseline. Higher scores on the x-axis indicate more positive contact with White individuals.

We next sought to relate group differences in the neural response to White faces to intergroup contact with White individuals. We focused on these results because they were the only group differences that emerged from the behavior and brain data. Limiting the brain-behavior analyses to those that were associated with group differences was important to reduce the likelihood of false positives. Regarding brain activity, group differences only emerged in the right dlPFC and dorsal striatum. With respect to behavior, a main effect of group emerged on both the amount of contact and positive contact measures. The main effect emerged because White-Americans had more overall contact and more positive contact with both White and Black individuals than did any other group. However, because the pattern of activation in the dlPFC and dorsal striatum revealed race differences, we further narrowed our focus on behavior to contact measures that revealed a main effect of target race or interaction between target race and participant group. No interactions emerged, but there was main effect of target race for positive intergroup contact. This effect emerged because all participants reported having had more positive contact with White, versus Black, individuals. We calculated the correlations using the parameter estimates for White faces from the two brain regions where group differences emerged. See Table 3 for a complete list of the correlations between positive intergroup contact with White individuals and the neural response in right dlPFC and dorsal striatum to White male faces. Among White American participants, more positive contact (i.e., quality of contact) with White individuals was negatively correlated with the neural response in the right dlPFC to White faces, *r*(17) = −0.51, *p* = 0.03 (see Figure 5). The same correlation was not significant among Chinese–American participants, *r*(17) = −0.27, *p* = 0.29; or Chinese participants, *r*(16) = 0.03, *p* = 0.91. Positive contact with White individuals did not predict neural response in the right dorsal striatum among any group, *p*s > 0.29.

**Table 3:** Correlations among intergroup contact and neural responses to White male faces.

Variables	1	2	3	4
**White American**				
(1) Amount of contact		0.34	−0.15	−0.24
(2) Quality of contact			−0.51^∗^	−0.04
(3) R-dlPFC activation				0.31
(4) R-dorsal striatum activation				
**Chinese–American**				
(1) Amount of contact		0.21	−0.22	0.31
(2) Quality of contact			−0.27	0.04
(3) R-dlPFC activation				−0.01
(4) R-dorsal striatum activation				
**Chinese**				
(1) Amount of contact		0.67^∗^	−0.20	0.25
(2) Quality of contact			0.03	0.27
(3) R-dlPFC activation				0.20
(4) R-dorsal striatum activation				

*The sample size ranges from 17 (Chinese) to 18 (White American and Chinese–American) based on participant group. R, right; dlPFC, dorsolateral prefrontal cortex. ^∗^p < 0.05. For p-values and plots of each correlated reported here, see Supplementary Figures S1–S3.*

## Discussion

Several key findings emerged from the current study. Behaviorally, we demonstrated that White- and Chinese–American participants and Chinese participants had similar levels of stereotype knowledge and implicit bias about White and Black individuals. In contrast, White- and Chinese–American participants had higher quantity and quality of intergroup contact with White and Black individuals compared to Chinese participants. Our results also identified cultural differences in the neural response to male outgroup members in brain regions typically associated with cognitive control (right dlPFC, dorsal striatum, and vlPFC). Specifically, across all three regions, White American participants demonstrated the expected attenuated neural response in ingroup (White) versus outgroup (Black) male faces. In fact, White American participants’ increased positive contact with their ingroup correlated with their attenuated response in the right dlPFC. Conversely, Chinese participants had heightened neural activation in these regions for White versus Black faces, and Chinese–American participants did not have differential neural response to White and Black faces. Taken together, these findings suggest that culture shapes the neural response that may be associated with cognitive control to outgroups.

The fact that the three groups did not differ in their stereotype knowledge or their implicit bias toward Black individuals suggests that awareness of stereotypes may not differentially affect prejudice in Eastern or Western cultures. Specifically, individuals from Eastern cultures who had relocated to the United States had similar levels of prejudice (i.e., IAT scores) toward Black individuals as was expressed by White American and Chinese–American participants who had lived in the United States their entire lives. Supporting this assertion, examining the strength of stereotypic associations by block of the IAT revealed that Chinese participants had similar strength of associations between Black individuals and negative words as White Americans or Chinese–Americans. This indicated that individuals in Eastern and Western cultures express similar levels of bias when they are exposed to similar stereotypes about multiple outgroups. This finding also raised the possibility that stereotype knowledge may be quickly acquired in new cultural contexts. Alternatively, the transmission of culture-specific stereotypes about relatively novel outgroups may occur before relocating to that culture – in this case, before Chinese participants relocated to the United States – for instance, through media portrayals of these groups (Weisbuch et al., 2009). Supporting the latter, prior work has shown anti-Black bias in Western and, to a lesser extent, Eastern cultures (Nosek et al., 2007). Our work may extend these findings by demonstrating that individuals from Eastern cultures had biases thought to be specific to other cultures. However, this interpretation is limited given that knowledge of stereotypes and bias about racial outgroups before Chinese participants relocated to the United States was not assessed.

In contrast to stereotype knowledge, intergroup contact with Black and White individuals varied among the participant groups, which was not a mutually exclusive possibility. As previously alluded to, stereotype information may be conveyed in multiple ways that are not limited by interpersonal experiences (e.g., through media portrayals; Weisbuch et al., 2009). Specifically, Chinese participants had significantly less amount and quality of intergroup contact with both White and Black individuals, relative to American participants. Moreover, this result persisted when comparing Chinese–American and Chinese participants, which suggests that race alone did not limit one’s social interactions with outgroup members. Instead, Chinese participants appear to have had fewer experiences because of their relatively much shorter time in the United States. Cultural differences in contact with outgroups have important implications for prejudice in two ways. First, because intergroup contact is perhaps one of the most effective interventions for reducing prejudice (Dovidio et al., 2003; Pettigrew and Tropp, 2006), less contact affords fewer opportunities to do so. Perhaps for this reason, less intergroup contact also predicts less acculturation (Ward and Kennedy, 1993), which includes having social interactions and friendships with outgroups (Barry, 2001). In fact, Chinese participants in our study reported less acculturation along dimensions reflecting close, positive intergroup connections (i.e., assimilation and separation; Barry, 2001). In turn, less acculturation among immigrants predicts greater stress and poorer mental health outcomes (Ward and Kennedy, 1993; Yeh, 2003).

Although the groups differed in the intergroup experiences they had previously had with Black and White individuals, our neuroimaging findings demonstrated that contact was correlated with White Americans’ response to White male faces in brain regions previously associated with cognitive control (Miyake et al., 2000; Kelly et al., 2004; Krendl et al., 2012; Amodio, 2014). This finding may reflect favoritism toward ingroup members, which contributes significantly to prejudice (Greenwald and Pettigrew, 2014). Supporting this possibility, more positive contact, rather than quantity of contact, predicted the attenuated dlPFC response to White faces among White American participants. This finding is consistent with prior work that has shown that the dlPFC is involved in maintaining regulatory responses (e.g., Wagner et al., 2001; Krendl et al., 2012; see also Amodio, 2014). However, we cannot discount the possibility that activation in the dlPFC may reflect more general social cognitive processes that is not limited to cognitive control. Therefore, future research should investigate whether the specific function of the dlPFC in maintaining controlled responses relates to ingroup favoritism and if it does so across cultures.

It is interesting to note that Chinese participants had increased activation to White versus Black faces in these regions. This pattern was not the same among Chinese–Americans, who had similar levels of activation to White and Black faces. Chinese–American participants also did not have the same pattern of neural activation as did White American individuals, as predicted. Speculatively, one possibility for the disparate patterns of neural activity for Chinese–Americans and Chinese participants may be due to the fact that Chinese–Americans perceived two outgroups (White and Black) from their own culture, and Chinese participants perceived two outgroups from a different culture. Chinese–Americans had less self-reported contact (both amount and quality) with White and Black individuals than did White Americans. Thus, it is possible that their similar neural response to two outgroups from their same culture may be attributed to having had relatively less contact with each. However, because Chinese participants were perceiving two outgroups from different cultures, culturally-specific reasons may explain why Chinese participants exhibited a differentiated neural response to Black and White faces. For instance, White individuals have higher status (Penner and Saperstein, 2008) in the United States compared to Black individuals. Considering that Eastern cultures emphasize group differences to a greater extent than Western cultures (Triandis and Trafimow, 2003), Chinese participants’ increased activation in regions associated with cognitive control may reflect the relative status of White versus Black individuals in the United States. Based on the cultural differences associated with intergroup contact described above, one possibility is that Chinese participants required more cognitive resources when perceiving a higher status outgroup with whom they are more likely to interact, whereas Chinese–American participants did not. However, because no measures of perceived status of White and Black individuals were collected in the current study, we cannot address whether the likelihood of contact or status of Whites in Chinese participants’ current cultural context (the United States) may explain the heightened neural response between the outgroups.

A limitation of the current study is that the stimuli did not allow us to examine neural activation in response to ingroup faces for Chinese–American and Chinese participants (i.e., Chinese faces). For instance, some prior research found increased neural activations to same- versus other-race faces (Chiao et al., 2008; Adams et al., 2010), but other work found increased activations to other- versus same-race faces (Lieberman et al., 2005; Derntl et al., 2009). Moreover, cultural differences have been shown in the neural activation in response to same- versus other-race faces (Krendl, 2016), including in regions typically associated with affective aspects of stereotyping (Amodio, 2014) such as the amygdala and OFC (Chiao et al., 2008; Derntl et al., 2012). Thus, it is possible that cultural differences in neural activations related to stereotype knowledge may emerge more clearly when outgroup faces are contrasted against ingroup faces. Another important consideration when interpreting this work is that the cultural differences in neural activations were specific to male, versus female, outgroup faces. This pattern of results is consistent with prior work demonstrating that stereotypes about Black individuals, for instance, are more descriptive of men than women (Eagly and Kite, 1987; Johnson et al., 2012). However, in the current work, the measures of stereotypes and interpersonal experiences with outgroup members did not specify gender. This is a limitation because prior work found that gender plays an important role in both interracial interactions (Toosi et al., 2012) and stereotypes and meta-stereotypes (Babbitt et al., 2018). For example, White women are seen by both Whites and Blacks as less prejudiced than White men (Babbitt et al., 2018). Future research should investigate whether the cultural differences in awareness of stereotypes and stereotype content might depend not only on the target’s gender, but also the perceiver’s gender.

Despite these limitations, the current study provides useful insight into the cultural influences that contribute to how different outgroups, specifically male outgroup members, are stigmatized. While other work has shown cultural differences in neural activity associated with affect in response to outgroups (e.g., Chiao et al., 2008; Derntl et al., 2009, 2012), these findings provide new support that there are also cultural differences in the neural response associated with cognitive control to specific outgroups. Moreover, by examining this question within relatively novel cross-cultural context (i.e., among Chinese participants) we were able to characterize the influences of cultural learning about stereotypes and intergroup contact on the neural response to outgroups. Responses toward specific outgroups seems to emerge in a culturally-dependent way based on variability in intergroup contact, but this is not necessarily the case for stereotype knowledge or the strength of stereotypic associations (implicit bias). Altogether, these findings highlight the value of taking a cross-cultural perspective to the study of the factors underlying neural responses to outgroups.

## Ethics Statement

This study was carried out in accordance with the recommendations of the Institutional Review Board at Tufts University with written informed consent from all subjects. All subjects gave written informed consent in accordance with the Declaration of Helsinki. The protocol was approved by the Institutional Review Board at Tufts University.

## Author Contributions

AK and LB designed and performed the experiment. CH analyzed the data and wrote the manuscript with critical input from all authors.

## Conflict of Interest Statement

The authors declare that the research was conducted in the absence of any commercial or financial relationships that could be construed as a potential conflict of interest.

## Footnotes

1 www.fil.ion.ucl.ac.uk/spm
