# Supplementary material for: Culture Impacts the Neural Response to Perceiving Outgroups Among Black and White Faces
Source: Front Hum Neurosci. 2019 May 1;13:143. doi: 10.3389/fnhum.2019.00143 (PMC6504693; doi:10.3389/fnhum.2019.00143)
Supplement: Supplementary file 1 [file Table_1.DOCX]

***Supplementary Material***

**1 Supplementary Figures**


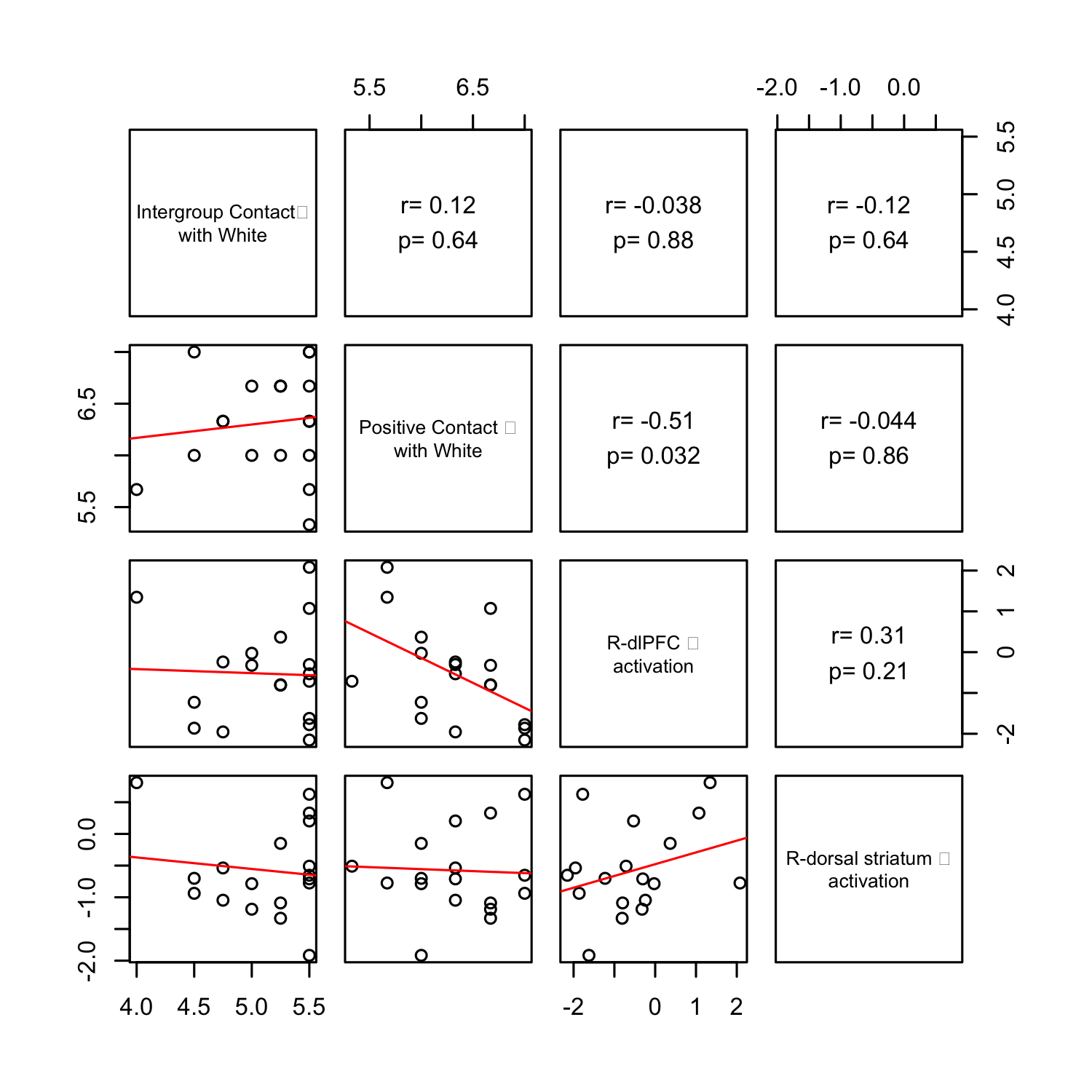


**SI Figure 1.** White American participants: Correlations among intergroup contact and neural responses to White male faces. *N* = 18. R = right. dlPFC = dorsolateral prefrontal cortex.


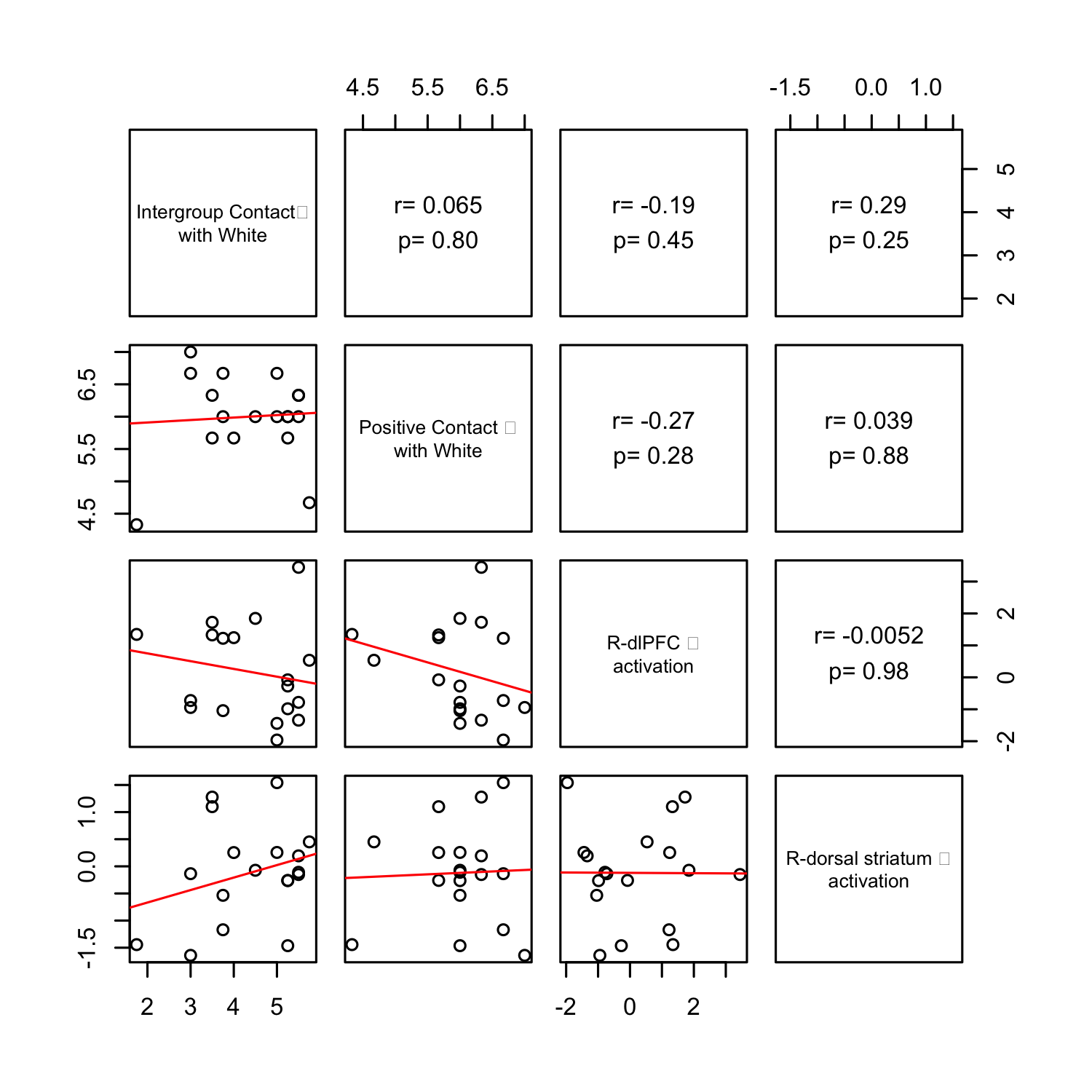


**SI Figure 2.** Chinese American participants: Correlations among intergroup contact and neural responses to White male faces. *N* = 18. R = right. dlPFC = dorsolateral prefrontal cortex.


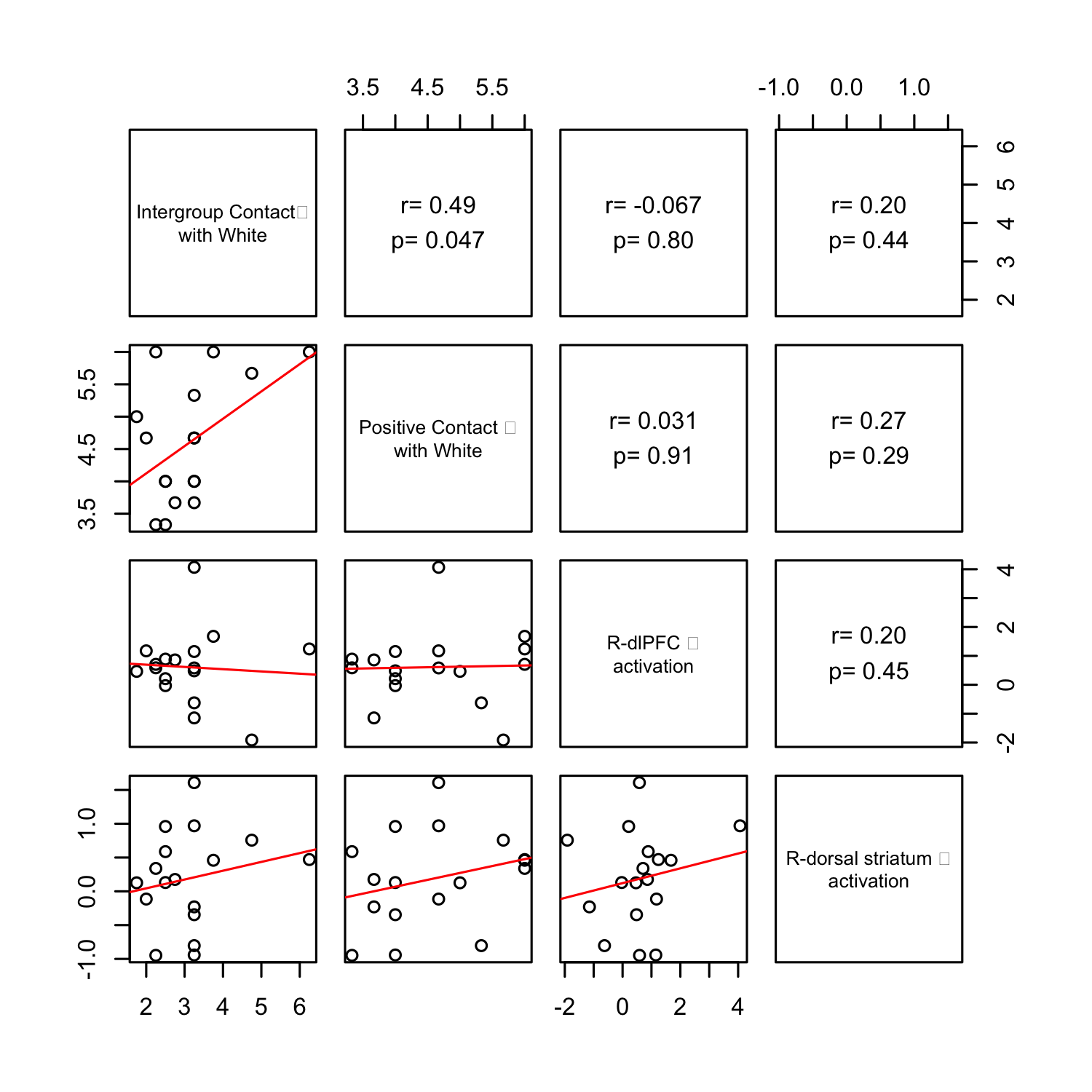


**SI Figure 3.** Chinese participants: Correlations among intergroup contact and neural responses to White male faces. *N* = 17. R = right. dlPFC = dorsolateral prefrontal cortex
